# Comparative Study on the Regeneration of Fe_3_O_4_@Graphene Oxide Composites

**DOI:** 10.3389/fchem.2020.00150

**Published:** 2020-02-28

**Authors:** Zhongliang Hu, Xiaojing Zhang, Jingying Li, Yirong Zhu

**Affiliations:** Department of Inorganic Nonmetallic Material, College of Metallurgy and Material Engineering, Hunan University of Technology, Zhuzhou, China

**Keywords:** graphene oxide, regeneration, Fe_3_O_4_, core-shell structure, magnetic graphene based composites

## Abstract

In this study, two kinds of composites with the structure of graphene oxide (GO) sheets wrapped magnetic nanoparticles were investigated on their regeneration. The composites have a similar core-shell structure, but the interactions between the core and shell are quite different, which are electrostatic and covalent. They were characterized by scanning/transmission electron microscopy, power X-ray diffraction, and vibrating sample magnetometer analysis. Their morphologies and structures of the samples had been revealed using methylene blue and Pb(II) as adsorbates during regeneration. The results showed that the composites with covalent bonding interaction could maintain a stable core-shell structure and present a good regeneration performance for adsorption-desorption of methylene blue and Pb(II). The composites with electrostatic interaction could approximately preserve its core-shell structure and could be recyclable for adsorption-desorption of methylene blue, however, they would disintegrate its core-shell structure during adsorption/desorption of Pb(II), thus greatly decreasing their regeneration performance. The regeneration mechanisms of the composites were analyzed, which could provide a useful theoretical guide to design the GO sheets wrapped magnetic nanoparticles composites.

## Introduction

Due to the unique structure and excellent characteristics, graphene and its derivatives have attracted more and more interests in the scientific community (Geim and Novoselov, 2007; Smith and Rodrigues, 2015; Nandhanapalli et al., 2019). Recently, graphene-based composites have been largely investigated as adsorption materials, which displayed excellent performances for adsorption of heavy metal ions, organic and dyes (Mi et al., 2012; Sitko et al., 2013; Yoon et al., 2016; Liu et al., 2018; Li et al., 2019). Notably, magnetic graphene oxide (GO) composites have been regarded as promising adsorbents for water purification because they could be easily separated from solution under an external magnetic field, thus overcoming the limitation of GO's difficult separation in solution (Chandra et al., 2010; He et al., 2010; Ma et al., 2018). However, GO sheets tend to restack, thus inevitably reducing their surface area and adsorption capacity (Bourlinos et al., 2003).

To further improve the performance, some researchers have attempted to wrap Fe_3_O_4_ nanoparticles (NPs) with GO sheets and the obtained GO@Fe_3_O_4_ displayed excellent adsorption performance toward pollutants (Wei et al., 2012; Pan et al., 2016). Compared with the magnetic GO composites where Fe_3_O_4_ is deposited on GO sheets, the GO@Fe_3_O_4_ composites have two obvious advantages. Firstly, they have much more stable structure because the connection area between GO and Fe_3_O_4_ is much more extensive; secondly, the restacking of GO sheets could be completely avoided, thereby enhancing their performances.

We have synthesize magnetic GO composites as environmental materials recently (Hu et al., 2009, 2010, 2012). Fe_3_O_4_ NPs have been successfully encapsulated with GO sheets though electrostatic interaction and covalent bonding, and both of the GO-based composites exhibited excellent adsorption toward contaminants in solution (Hu et al., 2017, 2020). Further researches revealed that the two kinds of composites displayed quite different regeneration toward heavy metal ions and organics. It is known that good regeneration is the prerequisite for the commercial application of the GO-based adsorbents due to their relatively high cost. Therefore, the investigations on the regeneration of GO-based adsorbents are very meaningful for related researches. Unfortunately, there exist rarely systematic studies on the regeneration of GO-based composites. Previous surveys only involved their adsorption capacity after several cycles, but the associated regeneration mechanisms including the morphology and structure evolution during regeneration had been scarcely investigated.

In this paper, our investigations are focused on the regeneration processes and the mechanisms of two kinds of Fe_3_O_4_@GO composites, which had been successfully synthesized in our previous study. Although both of the Fe_3_O_4_@GO samples have similar a core-shell structure, in which GO sheets are tightly connected with magnetic NPs, the interactions linking the core and shell are quite different, which are electrostatic and covalent, respectively. Methylene blue and Pb(II) were used as typical adsorbates to elucidate the evolution of the morphologies and structures of both samples during regeneration in detail. To the best of our knowledge, it is firstly reported to systematically investigate the regeneration mechanisms of the Fe_3_O_4_@GO composites, and the study could provide a theoretical guide for improving the regeneration of GO-based composites, thus accelerating their practical application.

## Experimental

### Materials

Graphite (100 mesh, XFnano), ferric chloride hexahydrate (FeCl_3_·6H_2_O, Sinopharm), ethylene glycol (Sinopharm), polyethylene glycol (PEG 4000, Sinopharm), sodium acetate trihydrate (NaAc·3H_2_O, Sinopharm), tetraethyl orthosilicate (Sinopharm), poly(diallyldimethylammonium chloride) (PDDA, Sinopharm), 3-aminopropyl triethoxysilane (APTES, Sinopharm), 1-ethyl-3-(3-dimethylaminopropyl) carbodiimide hydrochloride (EDC, Sinopharm), n-hydroxysuccinimide (NHS, Sinopharm), ammonia water (28 wt.%, Sinopharm), hydrochloric acid (Sinopharm), lead nitrate [Pb(NO_3_)_2_, Sinopharm], methylene blue (MB, Sinopharm).

### Materials Synthesis

#### Synthesis of GO Wrapped Fe_3_O_4_ Composites by Electrostatic Interaction

The synthesis procedures of GO wrapped Fe_3_O_4_ composites by electrostatic interaction (Fe_3_O_4_@GO-e) were described elsewhere in detail (Hu et al., 2020). The preparation steps included synthesis of Fe_3_O_4_, SiO_2_ coating on Fe_3_O_4_, introduction of PDDA on Fe_3_O_4_ and encapsulation of GO's sheets on magnetic particles. SiO_2_ coating endowed Fe_3_O_4_ with rich hydorphilic groups such as –OH, which can attract PDDA molecules with positive charges, and the positive charges on Fe_3_O_4_ could induced the coating of GO's sheets with negative charges, forming a core-shell structure. In brief, solvothermal synthesized Fe_3_O_4_ NPs were coated with a layer of SiO_2_ by a modified Stoker method (Gao et al., 2013), then the surface modified Fe_3_O_4_ NPs were mixed with PDDA solution. At the same time, the graphite oxide prepared by Hummers' method (Hummers and Offeman, 1958) was dispersed in distilled water. The dispersed GO solution was mixed with the above solution, and reacted for 8h. After washing, separation and drying, Fe_3_O_4_@GO-e was obtained.

#### Synthesis of GO Sheets Wrapped Fe_3_O_4_ Composites by Covalent Bonding

The detailed synthesis processes of GO sheets wrapped Fe_3_O_4_ composites by covalent bonding (Fe_3_O_4_@GO-c) could be found elsewhere (Hu et al., 2017), and they were composed of the following steps: synthesis of Fe_3_O_4_, SiO_2_ coating on Fe_3_O_4_, amination surface on Fe_3_O_4_ and final coupling reaction between GO and Fe_3_O_4_. The former procedures of solvothermal synthesis of Fe_3_O_4_ NPs and subsequent SiO_2_ coating were same as those of GO@Fe_3_O_4_-e except for some parameter modifications. After SiO_2_ coating, the amination on the surface of the magnetic NPs was carried out using APTES precursor, thus endowing the magnetic particles with rich amino groups on their surface. Meanwhile, the graphite oxide prepared by Hummers' method was dispersed in distilled water and its pH value was adjusted with a buffer solution. Subsequently, EDC and NHS were added into the GO solution, and finally the mixed solution reacted with aminated Fe_3_O_4_ NPs, resulting in the formation of Fe_3_O_4_@GO-c.

### Materials Characterization

Scanning electron microscopy (SEM) images were obtained using a JEOL JSM-6360LV or Hitachi S4800. Transmission electron microscope (TEM, Tecnai G2 F20, FEI, USA) was utilized to investigate the morphology and microstructure of the sample. The powder X-ray diffraction (XRD) patterns of the samples were collected from a Bruker D8 advanced diffractometer using Cu-Kαradiation (λ = 0.1514 nm) in the 2θ range of 10–80°. The magnetic experiments were performed on a Lakeshore 7407 vibrating sample magnetometer at room temperature.

### Adsorption Experiments

MB, a common dye pollutant, and Pb(II), a typical heavy metal ion, were used as adsorbates for the study. The adsorption experiments were carried out on a shaker with a shaking speed of 200 rpm at 30°C.

For MB adsorption tests, 50 mg of the sample and 50 mL of MB solution (150 mg/L, pH = 8) were mixed in a 100 mL air-tight glass conical flask. The adsorption equilibrium was reached after 2 h of agitation. Subsequently, the adsorbent was separated using a hand-held permanent magnet. The supernatant was collected for concentration measurements by UV-vis spectrophotometry. The adsorption capacity was calculated based on the following formula:

(1) $$\begin{array}{l} q_{e}=\frac{\left(C_{0}-C_{e}\right)V}{M} \end{array}$$

where q_e_ refers to the adsorption equilibrium capacity, C_0_ and C_e_ denote the initial and equilibrium concentrations, respectively, V is the solution volume, and M represents the adsorbent's mass.

For Pb(II) adsorption, the experimental procedures were same as the above ones except for modifications of the following parameters. The initial concentration of Pb(II) solution was 300 mg/L, and its pH value was adjusted to 6 before adsorption tests. The adsorption equilibrium time was set as 12 h. The Pb(II) concentration in the supernatant was measured by atomic absorption spectrophotometry and the adsorption capacity toward Pb(II) was obtained according to Equation (1).

### Desorption and Regeneration Experiments

The MB-loaded and Pb(II)-loaded samples were utilized to evaluate the regeneration performance. For MB desorption, the regeneration of the sample was carried out by immersing it in ethanol solution under mechanical stirring for 30 min. For Pb(II) desorption, the regeneration of the sample was performed by soaking it in the presence of 0.01 M of HCl under ultrasonication for 30 min.

## Results and Discussions

### SEM/TEM Analyses of the Fe_3_O_4_@GO Samples

**Figure 1:**
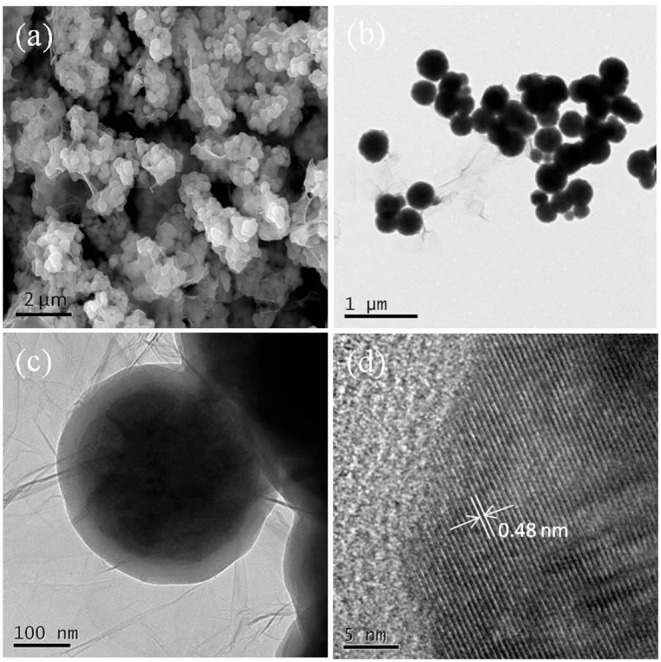
**(a)** SEM image of Fe_3_O_4_@GO-e, **(b)** TEM image of Fe_3_O_4_@GO-e, **(c)** magnified TEM image of **(b)**, and **(d)** high-resolution TEM image of Fe_3_O_4_@GO-e.

SEM/TEM could intuitively reveal the morphologies and textures of the samples. The SEM/TEM images of the Fe_3_O_4_@GO-e sample are shown in Figure 1. From the SEM image (Figure 1a), it could be clearly observed that the Fe_3_O_4_ NPs or Fe_3_O_4_ NP aggregations are tightly wrapped by the silk-like GO sheets. The TEM images of the sample (Figures 1b,c) further demonstrate that the corrugated GO sheets are compactly connected with the magnetic NPs. The high-resolution TEM of the sample is displayed in Figure 1d, in which the atomic lattice fringes could be distinctly observed. The interplanar spacing (~0.48 nm) could be attributed to the (111) lattice plane of the Fe_3_O_4_ crystal. In this study, the good structure of the Fe_3_O_4_@GO-e sample could be ascribed to the utilization of Fe_3_O_4_ NPs with large surface area, rich -OH groups on the surface introduced by SiO_2_ coating, and PDDA with positive charges which could strongly attract the negative GO sheets.

**Figure 2:**
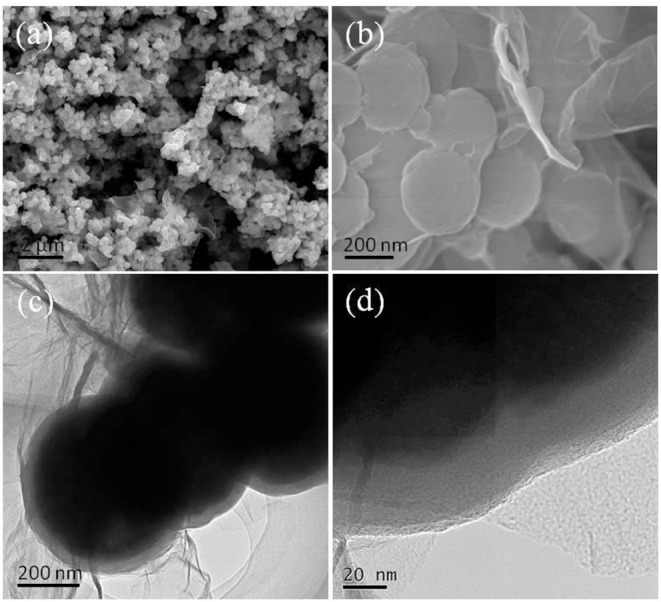
**(a)** SEM image of Fe_3_O_4_@GO-c, **(b)** magnified SEM image of **(a)**, **(c)** TEM image of Fe_3_O_4_@GO-c, and **(d)** magnified image of **(c)**.

Figure 2 shows the SEM/TEM images of the Fe_3_O_4_@GO-c sample. Compared with the Fe_3_O_4_@GO-e sample, Fe_3_O_4_@GO-c also presents a core-shell structure, where the magnetic NPs are roundly encapsulated by the wrinkled silk-like GO sheets. Its SEM image is similar to Fe_3_O_4_@GO-e's, indicating that the encapsulation effect of the sample is good as Fe_3_O_4_@GO-e's. However, the TEM images exhibit that the crinkly GO sheets are more densely and firmly grafted to the magnetic NPs surface, inferring that the sample has a more stable structure than Fe_3_O_4_@GO-e. The structure difference between two samples could be reasonably deduced that the covalent bonding in Fe_3_O_4_@GO-c is much stronger than the electrostatic interaction in Fe_3_O_4_@GO-e. This difference could have a significant influence on their regeneration.

### Morphology and Structure Analyses of the Fe_3_O_4_@GO Samples After Regeneration

**Figure 3:**
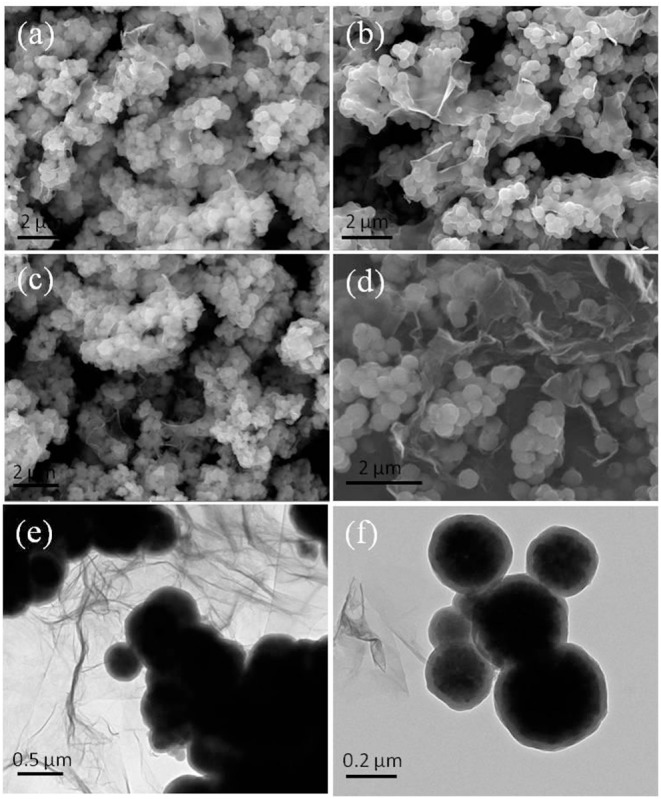
SEM images of the samples after five cycles **(a)** Fe_3_O_4_@GO-c toward MB, **(b)** Fe_3_O_4_@GO-e toward MB, **(c)** Fe_3_O_4_@GO-c toward Pb(II), and **(d)** Fe_3_O_4_@GO-e toward Pb(II); TEM images of the samples after five cycles **(e)** Fe_3_O_4_@GO-c toward Pb(II) and **(f)** Fe_3_O_4_@GO-e toward Pb(II).

The regenerability of the sample has much to do with its evolution of morphology and structure during adsorption-desorption recycling. Figure 3 displays the SEM/TEM images of the samples after five cycles of adsorption-desorption toward MB and Pb(II). Compared with the morphology before adsorption test, the morphology of Fe_3_O_4_@GO-c changed little (Figures 3a,c,e), indicating that it could maintain its structure during regeneration toward MB and Pb(II). As a result, it could be recyclable. The next tests of adsorption-desorption further demonstrate that the stable structure is beneficial to enhancing its regenerability.

Nevertheless, the Fe_3_O_4_@GO-e sample had quite different morphologies and structures after adsorption-desorption recycling toward MB and Pb(II). From Figure 3b, it can be seen that the morphology and core-shell structure of Fe_3_O_4_@GO-e are approximately preserved, manifesting that the Fe_3_O_4_@GO-e sample could keep stable during regeneration toward MB. However, after adsorption-desorption toward Pb(II), its morphology and structure had completely changed. Figure 3d clearly shows that the magnetic NPs are not wrapped by GO sheets and instead they lie on GO sheets via weak connection. The TEM image (Figure 3f) further shows that the GO sheets have been almost separated from the magnetic NPs. Obviously, the Fe_3_O_4_@GO-e sample had disintegrated and its core-shell structure had been destroyed.

### XRD and Magnetic Property Analyses

**Figure 4:**
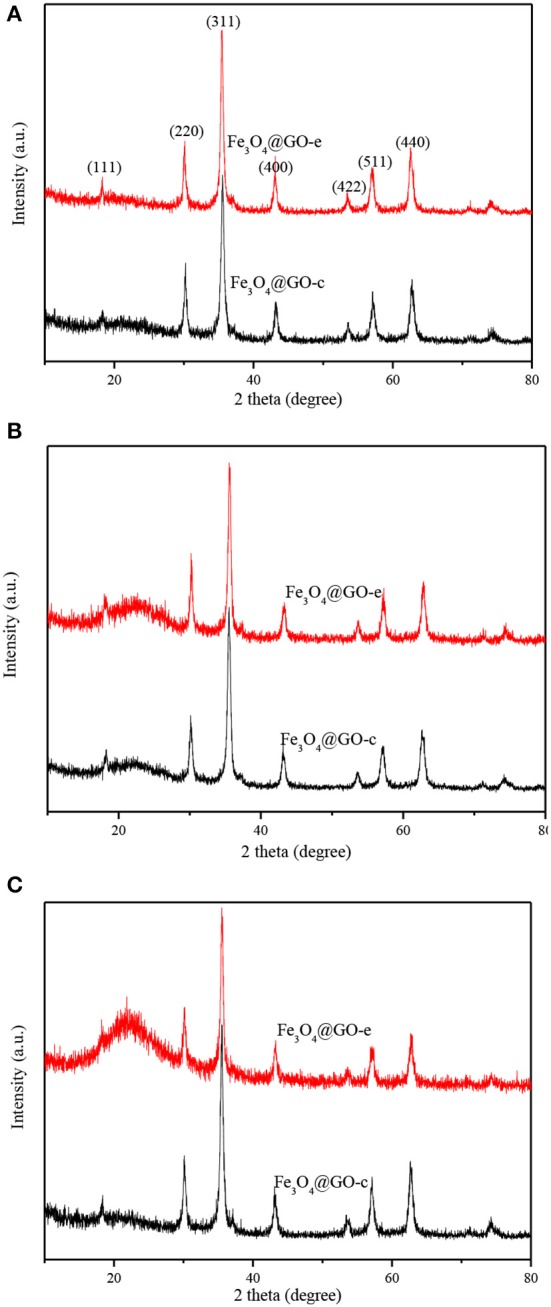
XRD patterns of Fe_3_O_4_@GO-e and Fe_3_O_4_@GO-c **(A)**, and Fe_3_O_4_@GO-e and Fe_3_O_4_@GO-c after five adsorption-desorption cycles toward MB **(B)** and Pb(II) **(C)**.

XRD technique is a powerful tool for structure characterization, and the structure variation of the Fe_3_O_4_@GO samples mainly lies in whether the GO sheets could still wrap the magnetic NPs or disintegrate from the NPs. The XRD patterns of Fe_3_O_4_@GO samples before and after recycling are shown in Figure 4. It is evident that all samples have the typical XRD pattern of magnetite (JCPDS No. 19-0629), indicating that the Fe_3_O_4_ NPs retain their original crystalline structure. It should be paid attention to the peaks variation at the 2θ range of 20–30° for the magnetic GO composites. The composites tend to appear with a broad XRD peak at 20–30° when metal NPs are deposited on the graphene sheets, which is due to the loose stacking of graphene sheets (Ai et al., 2011; Guo et al., 2015; Wu et al., 2016). However, in our case, there existed almost no peak at 20–30° for the Fe_3_O_4_@GO samples (Figure 4A). It could be explained that the peak at 20–30° is attributed to the stacking of the tiled GO sheets, which would disappear for the wrapped GO sheets. As for the regenerated samples, their XRD patterns are very different from the aboves. From Figure 4B, it could be seen that the regenerated Fe_3_O_4_@GO-c sample has a negligible peak whereas the regenerated Fe_3_O_4_@GO-e has a tiny peak at 20–30°. It could be deduced that the core-shell structure of Fe_3_O_4_@GO-c could be well maintained and Fe_3_O_4_@GO-e could generally preserve the core-shell structure during regeneration toward MB. However, after desorbing Pb(II), the Fe_3_O_4_@GO-e sample displays an intensive and broad peak at 20–30° (Figure 4C), manifesting that most GO sheets are separated from the magnetic NPs. On the contrary, the XRD peak of Fe_3_O_4_@GO-c are almost same as that in Figure 4A, verifying that it could well maintain core-shell structure during adsorption-desorption of Pb(II). The XRD results inherently reveal the structure evolution of Fe_3_O_4_@GO during adsorption-desorption process, which are highly consistent with the SEM/TEM characterizations. The results further clarify the structure evolution of the Fe_3_O_4_@GO samples during adsorption-desorption process.

The magnetism of novel carbon materials plays a pivotal role for their application as adsorbents (Ren et al., 2019; Zhang et al., 2019). The maximum saturation magnetizations of the GO-based samples are listed in Table 1, from which it could be seen that they change slightly after five cycles of adsorption-desorption toward MB or Pb(II). The negligible variation in magnetization can be deduced that the magnetism of the samples comes from Fe_3_O_4_ NPs and they are well-preserved in the samples after recycling. The good magnetization is very helpful to facilitate the post-processing of the GO composites. However, after desorption-desorption of Pb(II), the Fe_3_O_4_@GO-e sample has completely disintegrated its core-shell structure. As a result, a mixture of magnetic NPs and GO sheets was formed, therefore, it had to be separated from solution by membrane filtration instead of external magnet.

**Table 1:** The maximum saturation magnetisms of the Fe_3_O_4_@GO samples before and after five cycles of adsorption-desorption toward MB and Pb(II).

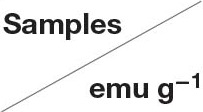	**No recycling**	**After five cycles toward MB**	**After five cycles toward Pb(II)**
Fe_3_O_4_@GO-e	61.0	59.5	59.1
Fe_3_O_4_@GO-c	57.1	55.8	55.3

### Regeneration Study

**Figure 5:**
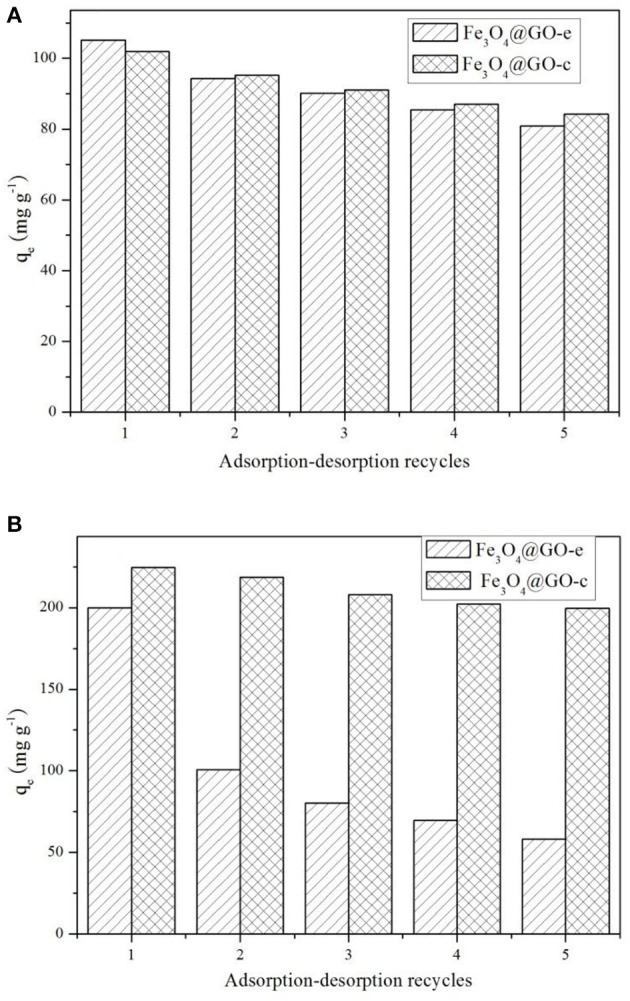
Adsorption-desorption recycling tests toward **(A)** MB and **(B)** Pb(II).

The adsorption-desorption tests for the samples were repeated five times using MB and Pb(II) as the adsorbates, and the results are displayed in Figure 5. Before recycling, Fe_3_O_4_@GO-e exhibits an adsorption capacity of 105.5 mg/g toward MB, which is a little higher than Fe_3_O_4_@GO-c (102.4 mg/g). However, Fe_3_O_4_@GO-c displays a greater initial adsorption amount (224.5 mg/g) than Fe_3_O_4_@GO-e (199.8 mg/g). It could be attributed to the fact that Fe_3_O_4_@GO-c is rich of –NH_2_ groups, which can help to chelate Pb(II). From Figure 5A, it could be observed that both of the Fe_3_O_4_@GO samples could maintain good regeneration even after five cycles toward MB, and the Fe_3_O_4_@GO-e and Fe_3_O_4_@GO-c samples could hold 77% and 83% adsorption capacities, respectively. Nevertheless, the samples present very different regeneration performances toward Pb(II). From Figure 5B, it could be seen that Fe_3_O_4_@GO-c could maintain ~89% adsorption capacity after five cycles whereas Fe_3_O_4_@GO-e only possesses ~29% adsorption capacity. Moreover, for the initial cycle, the adsorption capacity of Fe_3_O_4_@GO-e has been drastically decreased, inferring that its structure has greatly changed.

It is well-known that the performance of a composite is highly related to its structure. Therefore, it could be reasonably deduce that the significant variation of the Fe_3_O_4_@GO-e sample in adsorption capacity toward Pb(II) is attributed to the disintegration of its core-shell structure. Indeed, during desorption of Fe_3_O_4_@GO-e toward Pb(II), there existed black GO sheets in solution, which could not be separated by an external magnet, fully demonstrating that the GO sheets had been separated from the composites and dispersed in solution. The slight decrement in adsorption capacities in other cases could be explained that the pre-adsorbed amounts could not be totally released from adsorption sites (Zhang et al., 2013), hence verifying that their core-shell structures had been well-preserved. In this study, only Pb(II) was used for the investigation, but, as a typical heavy metal ion, the related results may be applied to other heavy metal ions because they had a similar adsorption mechanism by Fe_3_O_4_@GO-e and other GO-based adsorbents (Hu et al., 2017).

### Structure Evolutions of the Fe_3_O_4_@GO Samples During Regeneration Process

The structure evolution of the adsorbents during regeneration is closely related to their regeneration performance. Therefore, it is very beneficial to improving the structural stability by adoption of pertinent measures.

In this study, both Fe_3_O_4_@GO samples have a similar core-shell structure, in which the magnetic NPs are tightly wrapped by GO sheets. However, the interactions between the core and shell are completely different, which are electrostatic and covalent. The Fe_3_O_4_@GO-c sample has a stable structure due to the firm covalent bonding that could resist the acid and ethanol environment during regeneration, resulting in good regeneration performance toward MB and Pb(II). For the Fe_3_O_4_@GO-e sample, its structure is not very stable owing to the weak electrostatic connection, but it could still maintain its core-shell structure in ethanol solution, thus resulting in a reasonable regeneration performance toward MB.

**Figure 6:**
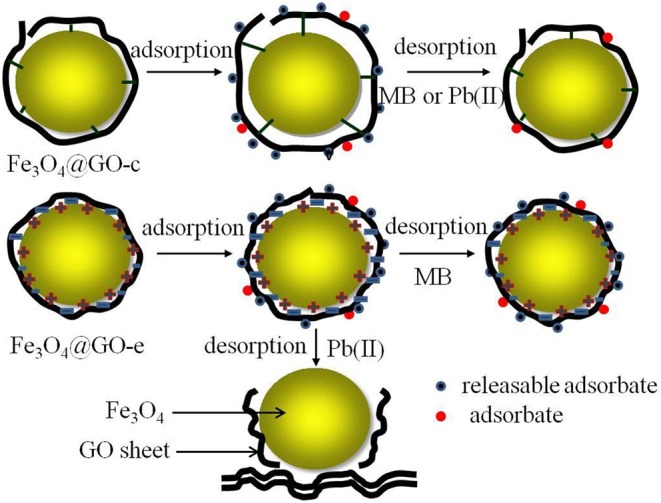
Sketch illustrating the structure evolution of Fe_3_O_4_@GO-c and Fe_3_O_4_@GO-e.

Unfortunately, when desorbing Pb(II) in acid solution, the Fe_3_O_4_@GO-e sample would disassemble due to the following reasons: (1) H^+^ ions could attract GO sheets with negative charges; (2) A great number of H^+^ ions in solution could endow GO sheets with positive charges, and it would repel the magnetic NPs with the same charges. The sketch illustrating the structure evolution during regeneration is presented in Figure 6.

## Conclusion

Two kinds of composites with the structure of GO sheets wrapped magnetic nanoparticles composites had been successfully synthesized, and their regeneration had been investigated using MB and Pb(II) as adsorbates. Both samples have a similar core-shell structure, and the linking forces between core and shell are electrostatic and covalent, respectively. During regeneration, the GO@Fe_3_O_4_-c sample could resist the erosion from ethanol and acid solution, and could well maintain its core-shell structure. After five cycles, it still holds the adsorption capacity ~83% toward MB and ~89% toward Pb(II), respectively. The GO@Fe_3_O_4_-e sample could preserve ~77% adsorption capacity toward MB, and could roughly keep the core-shell structure after five cycles. However, it would completely disassemble its core-shell structure, resulting in only ~29% adsorption capacity toward Pb(II) after five cycles. The related regeneration mechanism and the structure evolution during regeneration had been proposed, which could provide a theoretical guide for designing and improving the GO-based composites.

## Data Availability Statement

The datasets generated for this study are available on request to the corresponding author.

## Author Contributions

ZH and XZ conducted the most experiments. JL and YZ performed the characterization and data analysis. All authors involved the analysis of experimental data and manuscript preparation.

### Conflict of Interest

The authors declare that the research was conducted in the absence of any commercial or financial relationships that could be construed as a potential conflict of interest.
